# Effect of a Chronic Intake of the Natural Sweeteners Xylitol and Erythritol on Glucose Absorption in Humans with Obesity

**DOI:** 10.3390/nu13113950

**Published:** 2021-11-05

**Authors:** Valentine Bordier, Fabienne Teysseire, Götz Schlotterbeck, Frank Senner, Christoph Beglinger, Anne Christin Meyer-Gerspach, Bettina K. Wölnerhanssen

**Affiliations:** 1 St. Clara Research Ltd. at St. Claraspital, 4002 Basel, Switzerland; valentine.bordier@unibas.ch (V.B.); fabienne.teysseire@unibas.ch (F.T.); christoph.beglinger@unibas.ch (C.B.); 2 Faculty of Medicine, University of Basel, 4001 Basel, Switzerland; 3 Institute for Chemistry and Bioanalytics, School of Life Science, FHNW University of Applied Sciences and Arts Northwestern Switzerland, 4132 Muttenz, Switzerland; goetz.schlotterbeck@fhnw.ch (G.S.); frank.senner@fhnw.ch (F.S.)

**Keywords:** erythritol, xylitol, natural sweeteners, glucose absorption, 3-OMG, obesity

## Abstract

In patients with obesity, accelerated nutrients absorption is observed. Xylitol and erythritol are of interest as alternative sweeteners, and it has been shown in rodent models that their acute ingestion reduces intestinal glucose absorption. This study aims to investigate whether a chronic intake of xylitol and erythritol impacts glucose absorption in humans with obesity. Forty-six participants were randomized to take either 8 g of xylitol or 12 g of erythritol three times a day for five to seven weeks, or to be part of the control group (no substance). Before and after the intervention, intestinal glucose absorption was assessed during an oral glucose tolerance test with 3-*Ortho*-methyl-glucose (3-OMG). The effect of xylitol or erythritol intake on the area under the curve for 3-OMG concentration was not significant. Neither the time (pre or post intervention), nor the group (control, xylitol, or erythritol), nor the time-by-group interaction effects were significant (*p =* 0.829, *p =* 0.821, and *p =* 0.572, respectively). Therefore, our results show that a chronic intake of the natural sweeteners xylitol and erythritol does not affect intestinal glucose absorption in humans with obesity.

## 1. Introduction

Overweight and obesity are major public health problems of our century. They represent an enormous challenge in preventing chronic non-communicable diseases such as metabolic syndrome, cardiovascular diseases, and type two diabetes mellitus (T2DM) [1]. In obesity, many aspects of metabolism and energy homeostasis are dysregulated. For example, it has been shown that gastric emptying is delayed in adults with obesity compared to lean adults, and that their post-prandial increase in plasma glucagon-like peptide 1 (GLP-1) and peptide tyrosine tyrosine (PYY) is reduced [2]. Furthermore, in patients with obesity, the total number of gut enteroendocrine cells—cells responsible for satiation signal generation via hormone secretion—is significantly reduced in the stomach and duodenum, compared to lean subjects [3]. Additionally, patients with obesity often suffer from leptin resistance, which reduces satiation and thus increases energy intake [4,5]. It has also been shown that they develop an accelerated and more efficient absorption of nutrients and energy from meals than healthy participants [6]. In particular, intestinal glucose absorption is accelerated in patients with obesity [7]. This, combined with low incretins, promotes hyperglycemia and hyperinsulinemia. Indeed, the glucose and insulin responses to an intra-gastric glucose load (75 g) and to a mixed-nutrient drink are higher in patients with obesity compared to healthy participants [8,9].

Intestinal glucose absorption is mediated by the availability of intestinal sodium-dependent glucose cotransporter 1 (SGLT-1) and glucose transporter 2 (GLUT2) [10]. In health, the abundance of these transporters is upregulated in the short term by glucose and carbohydrates intake and by the secretion of hormones such as insulin, glucagon, and glucagon-like peptide 2, and in the long term by a high-carbohydrate diet [11]. In rats with induced diabetes, the glucose absorption is increased due to a higher number of SGLT-1 transporters [12]. In humans with non-insulin-dependent diabetes, SGLT-1 transporters were found to be 4.3 times higher and GLUT2 mRNA levels three times higher than in healthy subjects [13]. This higher glucose absorption in diabetes leads to exacerbation of hyperglycemic episodes [14]. Additionally, in patients with morbid obesity, a significant increase in SGLT-1 expression was also detected in the duodenum [3]. Therefore, intestinal glucose absorption is an essential aspect of the post-prandial glucose response, influencing overall caloric intake and glycemia, both of which are relevant in the etiology of obesity and T2DM.

Erythritol and xylitol, which are naturally occurring sugar alcohols, are of interest as alternative sweeteners. Besides their positive effects on caries [15], xylitol has been shown to have beneficial effects on visceral fat mass, plasma insulin, and lipid concentrations in a non-diabetic rat model [16], as well as on glycemia and diabetes [17,18] in a diabetic rat model. Erythritol, for its part, has been shown to have a protective effect on endothelial cell function, especially under hyperglycemic conditions, in experimental models as well as in diabetic patients [19,20]. Moreover, the acute ingestion of xylitol and erythritol induces the secretion of GLP-1, PYY, and cholecystokinin (CCK) without affecting insulin and plasma glucose concentrations and slows down gastric emptying [21,22,23].

Deane et al. noted that glucose absorption is lower when GLP-1 levels are high. The authors suggested that high GLP-1 levels slow down gastric emptying, which leads to a decreased intestinal glucose absorption and therefore a lower post-prandial glycemia [24]. The resulting hypothesis that erythritol and xylitol (which induce GLP-1 secretion) might reduce glucose absorption has been confirmed in a rodent model by Chukwuma et al. They were able to show that feeding rats with erythritol or xylitol (a single oral dose of 1 g/kg body weight) delayed gastric emptying and reduced small-intestinal glucose absorption in healthy (xylitol) and type-2 diabetic rats (both xylitol and erythritol) [25,26]. Additionally, a study with perfusion of xylitol combined with glucose in the small intestine of rats also showed a decrease in glucose absorption [27]. However, human studies on the effect of erythritol and xylitol on glucose absorption are lacking. Therefore, our study aimed to investigate whether a chronic intake of xylitol and erythritol would impact glucose absorption during an oral glucose tolerance test in humans with obesity but without diabetes. We hypothesized that chronic consumption of these natural sweeteners might positively affect glucose absorption and that therefore they might be promising sugar alternatives, combining caloric reduction with obesity and diabetes prevention.

## 2. Materials and Methods

### 2.1. Study Approval

This study is a secondary endpoint analysis of two different clinical trials having the same intervention (except for intervention durations of 5 and 7 weeks for trial 1 and trial 2, respectively) in the same participant population. The local ethics committee of Basel (Ethikkommission Nordwest- und Zentralschweiz; Trial 1: EKNZ 2016-00781; Trial 2: EKNZ 2016-00782) approved both trials, which were performed in compliance with the current version of the Declaration of Helsinki, the ICH-GCP, and national legal and regulatory requirements. Each participant gave written informed consent for trial participation. Both trials were registered at ClinicalTrials.gov (trial 1: NCT02821923; trial 2: NCT02824614).

### 2.2. Participants

A total of 46 participants with obesity but without diabetes (glycated hemoglobin (HbA1C) < 6.5%) (20 women, mean body mass index (BMI): 35.5 kg/m^2^, range: 30.4–45.6 kg/m^2^, mean age: 30.2 years, range: 18–54 years) took part in this study. Thirty-three participants were included in the first trial (trial 1), and 13 were included in the second trial (trial 2). The inclusion of participants occurred consecutively, after an amendment for the introduction of 3-*Ortho*-methyl-glucose (3-OMG) as a marker of glucose absorption was authorized by the ethics committee in both trials. For both trials, subjects were excluded if they suffered from chronic diseases or diseases of the gastrointestinal tract, if they had a history of surgery with major changes to the gastrointestinal tract, if they took medications regularly, if they were pregnant or if they were substance abusers. None of the participants had a history of dietary restrictions or pre-existing regular consumption of xylitol or erythritol.

### 2.3. Study Design

Both trials were conducted as randomized controlled trials. Participants were randomly assigned to one of the two intervention groups (consumption of either xylitol or erythritol every day for five weeks for trial 1 and seven weeks for trial 2) or the control group (no substance). In the intervention groups (but not in the control group), the trials were double-blinded, meaning that the study participant and the person who carried out all tests, as well as the personnel performing the analyses of blood samples, were blinded concerning the type of substance assigned to the participant.

### 2.4. Experimental Procedure

During the intervention period of five or seven weeks, participants in the intervention groups were asked to consume pre-portioned sticks of either 8 g of pure xylitol or 12 g of pure erythritol dissolved in water three times per day, before the main meals. Participants in the control group did not consume any substance. The dosage of erythritol was determined based on two considerations. Firstly, a pilot trial by Flint et al. showed metabolic effects after the chronic intake of 36 g of erythritol per day [20]. Secondly, the total daily sugar consumption in Switzerland is estimated to be ca. 120 g per day, of which 20% (i.e., ca. 24 g) is consumed as added sugar. The chosen quantity of erythritol is a feasible dosage to replace the daily added sugar intake and was chosen to represent real-life conditions. Xylitol was given in an equisweet dosage to erythritol.

The first week was an adaptation week, starting with one single portion per day before breakfast for two days, then two portions per day before breakfast and before lunch for three days, and finally three portions per day for two days. Participants went on with three portions per day for the following four and six weeks in trial 1 and trial 2, respectively.

Before and after the intervention period, all participants were admitted to St. Clara Research Ltd., Basel, Switzerland, in the morning after an overnight fast, for an oral glucose tolerance test (OGTT). For this purpose, they were instructed to abstain from strenuous exercise for the two days before the test, to make sure that their main meals contained carbohydrates, and to attend on an empty stomach, i.e., not to eat or drink (water allowed until two hours before the test) nor to consume alcohol within the ten hours before the test.

An antecubital catheter was inserted into a forearm vein for blood collection. After taking a fasting blood sample (t = −15 min), participants received a standardized glucose solution containing 75 g of glucose and 3 g of 3-OMG. 3-OMG is a non-metabolizable glucose analog used as a marker of glucose absorption. At regular time intervals after administration of the solution (t = 30, 60, 90, and 120 min), blood samples were taken for the analysis of plasma 3-OMG.

### 2.5. Blood Sample Collection and Processing

Blood samples were collected on ice into tubes containing ethylenediaminetetraacetic acid (EDTA). After centrifugation (4 °C at 3000 rpm for 10 min), plasma samples were processed into aliquots. The samples were then stored at −80 °C until they were analyzed.

### 2.6. Materials

Xylitol and erythritol were purchased from mithana GmbH (Zimmerwald, Switzerland); 3-OMG was purchased from Sigma-Aldrich (Merck, Kenilworth, NJ, USA).

### 2.7. Assessment of 3-OMG Concentrations

The plasma samples were extracted with a solution of acetonitrile, isopropanol, and water (3/3/2) and dried at 55 °C in a vacuum centrifuge for one hour. Before analysis, they were derivated with pyridine/N-Methyl-N-(trimethylsilyl)-trifluoracetamide at 37 °C. The concentration of 3-OMG in plasma was then assessed using gas chromatography mass spectrometry with helium. Mannitol was used as internal standard.

### 2.8. Statistics

This study is a secondary endpoint analysis. Therefore, no sample size calculation was made, and a minimum number of 15 participants per group was chosen for reasons of comparability and practicability.

Participants’ characteristics (demographic variables and biochemical parameters) were compared between the two trials with unpaired *t*-tests and between the groups with one-way analysis of variance tests. For 3-OMG concentration profiles, incremental values were used to calculate the area under the curve (iAUC) using the trapezoidal rule. All datasets were tested for normality with the Shapiro–Wilk test. The mean iAUC values at 120 min (iAUC120) were compared between the two trials with unpaired *t*-tests. The differences in iAUC120 before and after intervention, as well as those between the groups, were determined using a repeated measures general linear model. In this model, time (pre or post intervention) was introduced as a within-subject effect and group (control, xylitol, or erythritol) as a between-subjects effect.

All statistical analysis was done using the statistical software package IBM SPSS Statistics for Windows, Version 27.0 (Armonk, NY, USA: IBM Corp.). Values are reported in the tables and displayed in the figures as mean ± standard error of the mean (SEM). Differences were considered to be statistically significant when *p <* 0.05. The Bonferroni correction for multiple testing was applied when appropriate and when significant results were found.

## 3. Results

All subjects tolerated the study well, and there were no adverse events that led to study discontinuation. There was one drop-out in the first trial. The participant withdrew her consent before the first study day (but after screening) without giving further details of the reasons for discontinuation. She was replaced. Complete data from the final 46 participants were available for analysis

### 3.1. Participants’ Characteristics

The demographic characteristics and biochemical parameters of the participants in each intervention group (control, xylitol, and erythritol) are presented in Table 1. The demographic characteristics were not significantly different between the two trials (age = 30.8 ± 1.6 years (trial 1) versus age = 28.7 ± 2.1 years (trial 2), *p =* 0.467; BMI = 34.8 ± 0.6 kg/m^2^ (trial 1) versus BMI = 37.4 ± 1.4 kg/m^2^ (trial 2), *p =* 0.054; gender distribution = 39% women (trial 1) versus 54% women (trial 2), *p =* 0.385). Biochemical parameters were also not significantly different between the two trials (HbA1C = 5.32 ± 0.07% (trial 1) versus HbA1C = 5.40 ± 0.06% (trial 2), *p =* 0.729; triglycerides = 1.8 ± 0.26 mmol/L (trial 1) versus triglycerides = 1.4 ± 0.26 mmol/L (trial 2), *p =* 0.383; total cholesterol = 4.9 ± 0.21 mmol/L (trial 1) versus total cholesterol = 5.0 ± 0.27 mmol/L (trial 2), *p =* 0.734).

### 3.2. Glucose Absorption

The means of iAUC120 were not significantly different between the two trials in any of the groups (see Table 2). Therefore, the results were pooled and analyzed as a whole.

**Figure 1:**
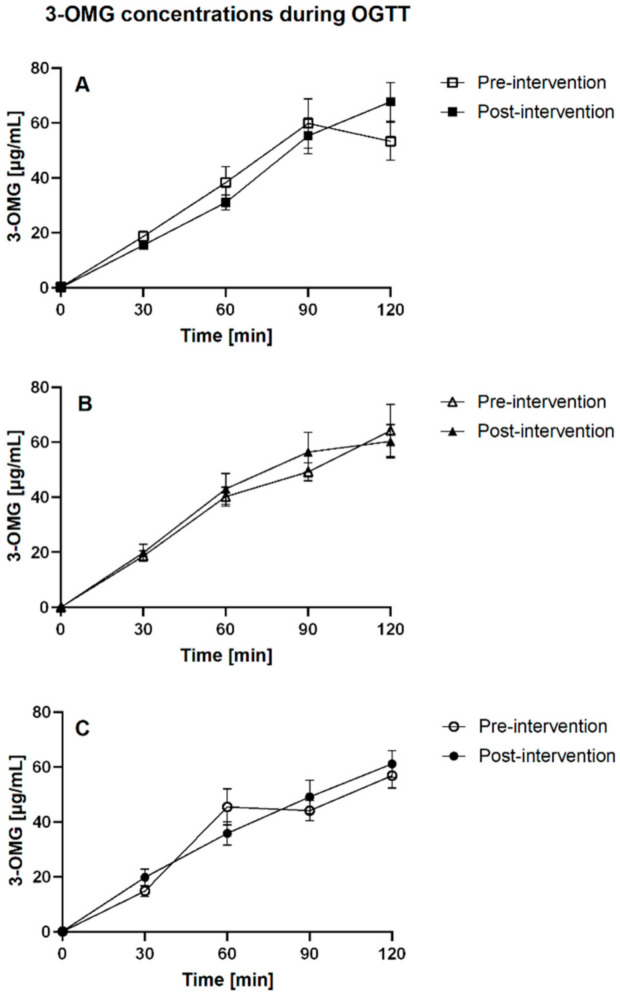
3-OMG concentrations before and after the intervention period in (**A**) the control group, (**B**) the erythritol group, and (**C**) the xylitol group. Data are displayed as mean ± SEM; OGTT: oral glucose tolerance test.

**Figure 2:**
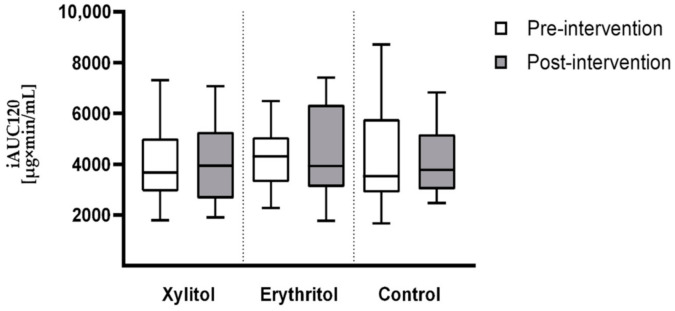
Boxplots of 3-OMG iAUC120 for each group before and after intervention. Data are displayed as median and inter-quartile range.

The effect of xylitol or erythritol intake on 3-OMG iAUC120 was not significant. Neither the time (pre or post intervention), nor the group (control, xylitol, or erythritol), nor the time-by-group interaction effects were significant (*p =* 0.829, *p =* 0.821, and *p =* 0.572, respectively, before Bonferroni correction). The means of 3-OMG iAUC120 for each group before and after intervention are reported in Table 3. The concentration curves are shown in Figure 1. Figure 2 displays the 3-OMG iAUC120 values before and after intervention for each group in the form of a boxplot.

## 4. Discussion

This study aimed to assess whether a chronic intake of xylitol or erythritol impacts intestinal glucose absorption during an oral glucose tolerance test (OGTT) in humans with obesity. The results show that the intake of xylitol or erythritol over a period of five to seven weeks does not significantly change glucose absorption during an OGTT compared to the baseline, and that glucose absorption after intake of either polyol is not different from the control group (no substance intake).

Our results are in contrast to the findings of Chukwuma et al., who showed that acute feeding of erythritol and xylitol in rats reduced intestinal glucose absorption [25,26], and to the results of Frejnagel et al., who reported reduced glucose absorption after small intestinal perfusion of xylitol and glucose in rats [27]. However, those studies were performed in animal models, and a direct extrapolation to the results in humans is not possible. More importantly, both groups assessed the effects of an acute dose of erythritol and xylitol, which differs from a chronic intake.

Other sweeteners have been investigated for their potential to alter glucose absorption. In a human trial, the effect of an intraduodenal infusion of sucralose combined with an intraduodenal infusion of glucose and 3-OMG was investigated. No effect of sucralose infusion on 3-OMG concentrations compared to a placebo infusion (saline) could be demonstrated [28]. Notably, the human trial was in contrast to the results of animal trials [29,30,31].

How can the results of the present study be explained? The regulation of glucose absorption depends in part on the availability of the two types of glucose transporters: SGLT-1 and GLUT2 [10]. While SGLT-1 is responsible for glucose absorption up to concentrations of about 30–50 mM of glucose in the lumen, GLUT2 is required to absorb glucose at higher concentrations [32]. Indeed, at concentrations of about 100 mM, GLUT2 is responsible for 56% of the absorption, compared to 27% at concentrations around 10 mM [33]. The GLUT2 transporters, which are located on the basolateral membrane of the enterocytes under low-sugar conditions, can be recruited to migrate to the apical membrane to augment the enterocytes’ absorption capacity [34,35]. This process is activated by the SGLT-1 transporters themselves [36], and it has been proposed that it is regulated by several mechanisms, such as calcium ion concentrations, sweet-taste receptor activation, and paracrine and endocrine hormones [32]. In health, insulin inhibits this process to prevent excessive glucose absorption after a sugar-rich meal. However, it has been observed that in insulin-resistant mice, this process is absent, and the levels of GLUT2 in the apical membrane remain constantly high [37]. Furthermore, in obesity, the glucose transporters are more numerous than in healthy persons [3]. Therefore, two possibilities may be considered, which might explain our findings: (1) the obese participants already had higher numbers of transporters to begin with, and the chronic intake of xylitol and erythritol did not affect their occurrence; (2) the recruitment of GLUT2 in the apical membrane, which happens within minutes after a sugar-rich meal, might be affected by an acute intake of xylitol or erythritol but was not seen during the OGTT performed after chronic intervention, without any preloading of the two substances immediately before the glucose administration. Therefore, acute studies are needed on the effect of xylitol and erythritol on glucose absorption in humans with and without obesity and diabetes, with observations of the glucose transporters in the intestine. A third explanation for our findings might be that the actual concentrations of xylitol and erythritol at the putative sites of action were too small to show improved metabolic effects in humans.

The study has some limitations. Firstly, this study was a secondary endpoint analysis; thus, no sample size calculation was performed. We can therefore not exclude the possibility that the study was underpowered to observe an effect of chronic xylitol and erythritol intake on glucose absorption. However, no reliable estimation for effect size was possible as human data are not available. We believe, however, that the current sample size provides clinically relevant information; an increase in the number of participants would be unlikely to change the conclusions. A second limitation concerns gastrointestinal hormones and glucose transporters. We did not measure GLP-1 or other gastrointestinal hormones during the OGTT, and neither did we take biopsies from the intestinal mucosa to quantify the presence and expression of the glucose transporters. Therefore, we can only speculate about the regulation of the mechanisms involved in glucose absorption after xylitol and erythritol intake. Thirdly, we did not assess gastric emptying rates, which might also help to clarify the action mechanisms. Fourthly, the design of this chronic study does not allow the investigation of acute effects. We cannot exclude the possibility that a decrease in glucose absorption occurred during the meals after xylitol and erythritol intake; however, this effect was not investigated. Fifthly, the study was double-blinded in the intervention groups only and not in the control group, as these participants did not consume any substance. However, there is no substance available that is equisweet with xylitol and erythritol and is without any known metabolic effects; therefore, a placebo control was not possible. Nevertheless, as participants served as their own controls (pre and post intervention), the placebo effect was mitigated. Finally, our study included patients with obesity but without diabetes. Studies on healthy normal-weight participants and patients with diabetes are warranted to better understand the regulation of glucose absorption in health and disease, and in relation to diet.

## 5. Conclusions

In conclusion, our results show that chronic intake of the natural sweeteners xylitol and erythritol does not affect intestinal glucose absorption in humans with obesity. This is of great interest for this specific patient population, as its members are at risk of complications such as T2DM and should therefore reduce their sugar consumption. This study assessed intestinal glucose absorption after an intake of the natural sweeteners that was very close to real-life conditions. Indeed, the dosage and the time points at which participants took the substances corresponded closely to how much and when added sugar is consumed in everyday life. The results imply that xylitol and erythritol can be consumed safely with regard to glucose absorption and can be used as sugar alternatives, even by patients with diabetes, as they do not affect glucose homeostasis.

## Figures and Tables

**Table 1:** Demographic characteristics and biochemical parameters (mean ± SEM (range)).

Parameter	Control Group	Xylitol Group	Erythritol Group	*p*-Value ^†^
**Gender**	*n* = 15 (8♀, 7♂)	*n* = 16 (4♀, 12♂)	*n* = 15 (8♀, 7♂)	0.191
**Age [yrs]**	31.2 ± 2.5	30.0 ± 2.2	29.4 ± 2.0	0.851
	(20; 54)	(19; 50)	(18; 39)	
**BMI [kg/m^2^]**	35.7 ± 1.1	35.2 ± 0.9	35.7 ± 1.1	0.933
	(30.3; 44.0)	(30.6; 44.8)	(30.7; 45.6)	
**HbA1C [%]**	5.4 ± 0.1	5.4 ± 0.1	5.3 ± 0.1	0.683
	(4.8; 5.7)	(4.7; 5.9)	(4.7; 6.4)	
**Triglycerides [mmol/L]**	1.8 ± 0.5	1.6 ± 0.2	1.8 ± 0.3	0.904
	(0.5; 8.0)	(0.4; 3.9)	(0.6; 6.1)	
**Total**	4.7 ± 0.3	4.8 ± 0.2	5.4 ± 0.4	0.163
**Cholesterol [mmol/L]**	(3.1; 7.2)	(2.8; 6.2)	(3.7; 9.6)	

^†^ One-way ANOVA, *p*-values before Bonferroni correction, ♀ stands for women, ♂ stands for men, BMI: body mass index, HbA1C: glycated hemoglobin.

**Table 2:** Comparison of 3-OMG iAUC120 (mean ± SEM) between the two trials.

Parameter	Group	Trial 1 (*n* = 33)	Trial 2 (*n* = 13)	*p*-Value ^†^
**3-OMG** **iAUC120** **[µg × min/mL]**	Xylitol pre	3753.56 ± 501.54	4485.24 ± 334.61	0.369
		(*n* = 11)	(*n* = 5)	
	Xylitol post	3910.68 ± 462.78	4382.42 ± 736.53	0.586
		(*n* = 11)	(*n* = 5)	
	Erythritol pre	4028.80 ± 423.00	4679.95 ± 163.88	0.385
		(*n* = 11)	(*n* = 4)	
	Erythritol post	4023.92 ± 462.40	5755.61 ± 933.62	0.09
		(*n* = 11)	(*n* = 4)	
	Control pre	4392.93 ± 680.42	4064.66 ± 812.92	0.796
		(*n* = 11)	(*n* = 4)	
	Control post	4261.61 ± 416.07	3560.07 ± 596.34	0.386
		(*n* = 11)	(*n* = 4)	

^†^ Unpaired *t*-tests; 3-OMG: 3-*Ortho*-Methyl-Glucose; iAUC120: area under the curve at 120 min.

**Table 3:** 3-OMG iAUC120 (mean ± SEM) for each group before and after intervention.

Parameter	Time Point	Control Group (*n* = 15)	Xylitol Group (*n* = 16)	Erythritol Group (*n* = 15)
**3-OMG** **iAUC120 [µg × min/mL]**	Pre-intervention	4305.39 ± 530.82	3982.21 ± 363.71	4202.44 ± 318.09
	Post-intervention	4074.53 ± 343.33	4058.10 ± 382.82	4485.70 ± 451.33

Neither the time, nor the group, nor the time-by-group interaction effects are significant (repeated measures general linear model).

## Data Availability

The data presented in this study are available on request from the corresponding authors.
